# Recommendations for Healthy Nutrition in Female Endurance Runners: An Update

**DOI:** 10.3389/fnut.2015.00017

**Published:** 2015-05-26

**Authors:** Louise Deldicque, Marc Francaux

**Affiliations:** ^1^Exercise Physiology Research Group, Department of Kinesiology, Faber, KU Leuven, Leuven, Belgium; ^2^Institute of Neuroscience, Université catholique de Louvain, Louvain-la-Neuve, Belgium

**Keywords:** iron, protein, carbohydrate, athlete, performance, oral contraceptives, calcium, vitamin D

## Abstract

The purpose of this review is to present the basic principles of a healthy nutrition in female endurance runner enriched by the latest scientific recommendations. Female endurance runners are a specific population of athletes who need to take specifically care of daily nutrition due to the high load of training and the necessity to keep a rather low body mass. This paradoxical situation can create some nutritional imbalances and deficiencies. Female endurance athletes should pay attention to their total energy intake, which is often lower than their energy requirement. The minimal energy requirement has been set to 45 kcal/kg fat free mass/day plus the amount of energy needed for physical activity. The usual recommended amount of 1.2–1.4 g protein/kg/day has recently been questioned by new findings suggesting that 1.6 g/kg/day would be more appropriate for female athletes. Although a bit less sensitive to carbohydrate loading than their male counterparts, female athletes can benefit from this nutritional strategy before a race if the amount of carbohydrates reaches 8 g/kg/day and if their daily total energy intake is sufficient. A poor iron status is a common issue in female endurance runners but iron-enriched food as well as iron supplementation may help to counterbalance this poor status. Finally, they should also be aware that they may be at risk for low calcium and vitamin D levels.

## Introduction

The importance of nutrition in endurance sports is obvious and the topic has already been nicely dealt in previous reviews (1–3). The purpose of the present work is to focus on specific recommendations that could be made for female endurance runners, a growing population that can reach up to 60% of the participants of a running event (http://www.runningusa.org/statistics). Indeed, more and more women are participating in running competitions and it may be assumed that this tendency will even grow in the next few years. It is therefore important to make appropriate nutritional recommendations taking the specific physiological requirements of female runners into account (4). The present review is purposely not exhaustive. It was rather chosen to highlight key and sensitive nutritional issues specific to the female endurance runner. The review will cover macronutrients requirements and will highlight the importance of three micronutrients for the female runner, i.e., iron, calcium, and vitamin D. Often overlooked, hydration will be addressed in one specific section. This review summarizes the information found on PubMed before April 2015 with the following search terms: female runner, female athlete, nutrition, diet, and hydration. The search was restricted to pre-menopausal and non-pregnant endurance runners.

## Low Energy Intake and Availability

Although running is a high-energy consuming physical activity, many endurance runners tend to keep their total energy intake very low to reduce body fat and weight (5). As running is a weight-bearing discipline, it is believed that the lighter the body weighs, the better the performance, which is far too simplistic and can lead to dramatic situations of leanness and nutritional deficiencies. While also present in male runners, the fight for a low percentage of body fat and a low body weight is most often observed in female athletes (6, 7). Likely because female runners need to challenge their body composition further from their natural shape than males to achieve the leanness that is considered optimal for the discipline. Trying to eliminate body fat beyond the biological disposition can have direct negative effects as for example disturbances of the adipose tissue secretome. Restricted energy, protein, carbohydrate, and micronutrients intakes are other more subtle and more indirect consequences from nutritional strategies aiming at decreasing weight and body fat (1), which may finally have significant effects on health and physical performance (8). Some athletes develop clear medical and/or psychological troubles such as eating disorders, osteopenia, and chronic menstrual dysfunction whereas others develop sub-clinical versions of these diseases (1). The expression “female athlete triad” encompasses the spectrum of restrained eating, menstrual dysfunction, and poor bone health from the lighter manifestation to the more severe. For those who are interested in this topic, we recommend the consensus statement published in 2014 (8, 9). A recent epidemiological study shows that the prevalence of sportswomen presenting simultaneously the three components of the female athlete triad is rather low, between 0 and 16%. However, those presenting one or two concurrent components, with different degrees of severity, approach 50–60% among certain athlete groups, including female endurance runners (10).

The physiological mechanisms leading to chronic energy deficit in female athletes are far from being elucidated but recent studies in exercising women with functional hypothalamic amenorrhea highlight the potential role of appetite-related hormones in the etiology of chronic energy deficiency and menstrual disturbances (10). Similarly to patients with anorexia nervosa (11), exercising women with functional hypothalamic amenorrhea exhibit paradoxically elevated levels of the orexigenic hormone, ghrelin, and the anorexigenic hormone, peptide YY, yielding a general anorexigenic effect (12, 13). Furthermore, fasting peptide YY concentrations have been negatively correlated with the rate of energy expenditure and positively correlated with a score of leanness in women ranked according to their level of physical activity and their menstrual status (10). High peptide YY and ghrelin concentrations, and the subsequent suppressive effect on appetite, suggest that peptide YY is able to blunt the orexigenic effects of ghrelin and prevents compensatory increases in energy intake in exercising women with functional hypothalamic amenorrhea. However, the impact of the quality of the diet and the amount of caloric intake on the menstrual cycle and bone mineral density have probably been overestimated. It is debatable whether the diet really influences the menstrual cycle in athletes who demonstrate low bone mineral density (4). Indeed, amenorrheic athletes have been shown to consume significantly fewer calories compared with eumenorrheic athletes (14) while similar energy intakes between the two groups have been reported as well (15). Specific nutrients, such as fiber, protein, calcium, colecalciferol, and vitamin K, have been shown to contribute to menstrual irregularities and low bone mineral density, also producing conflicting results (4, 16). Only little evidence exits up to now to support the hypothesis that dietary deficiencies, in particular calcium intake, favor osteoporosis in healthy recreational and professional athletes, even though abnormal and restrictive eating behaviors seem to be related to a larger probability of fractures (17).

From a practical perspective, the exact energy requirement of an athlete is not easy to quantify but a general consensus exists on the minimal energy availability needed for a female athlete (8). This is the energy required for living in a healthy way without taking the energy spent during physical activity into account. The energy available is therefore the total energy intake brought by nutrition minus the energy spent during physical activities. The target for an athlete should be at least 45 kcal/kg of fat free mass per day (Table 1). Concretely for an athlete of 50 kg having a fat percentage of 15% and a fat free mass of 42.5 kg, the energy available should be above 1900 kcal/day. Having an energy expenditure of 500–600 kcal during sport activities would increase the daily total energy intake to 2400–2500 kcal.

**Table 1:** **Specific nutritional recommendations for the female endurance runner**.

	Daily requirement	Remarks
Energy intake	>45 kcal/kg fat free mass + energy spent during physical activity	
Protein	1.2–1.4 g/kg	Recent research suggests 1.6 g/kg/day
Carbohydrate loading	>8 g/kg	Efficient if total energetic balance is adequate
Iron	18 mg	Somewhat lower for oral contraceptive users (11–12 mg/day)
Calcium	1000 mg	Amenorrheic athletes may require an additional 500 mg/day
Vitamin D	300–2000 IU	RDA is inversely related to sun exposure
		Serum 25(OH)D concentration should be above 75–80 nmol/l
Water	2 l + water lost during physical activity	Water found in beverages and food

*RDA, recommended dietary allowances*.

## Proteins

Acute endurance exercise results in the oxidation of several amino acids, which provides 1–6% of the total energy cost of exercise (3). With adequate energy and carbohydrate intake, low to moderate intensity endurance exercise has little impact on dietary protein requirements and 1 g protein/kg/day is sufficient (3). The only situations where dietary protein requirements have been proven to exceed the requirements for relatively inactive individuals are: (1) in elite male athletes where the maximal requirement is approximately 1.6 g protein/kg/day; (2) in case of low energy and/or low carbohydrate intake (3, 18). In this second situation, protein requirement is much more complicated to estimate. Unfortunately, this state of energy and/or carbohydrate deficit is not uncommon in female endurance runners as mentioned above. Protein requirements for elite endurance athletes have mainly been calculated in men but it seems that requirements for women are about 25% lower than those for men, i.e., 1.2–1.3 g protein/kg/day (19, 20). Most athletes are able to reach these protein requirements from their usual daily diet as long as proteins represent 10–15% of the energy and as long as total energy supply is adequate (3). Nevertheless, protein intake should be assessed on grams per kilogram basis instead of a percentage of the diet, as the latter could result in low absolute intakes in energy restricting athletes.

Very recently, the first empirical measurement of nitrogen turnover was made in endurance trained female athletes (21). The estimated daily protein requirement to maintain nitrogen balance was 1.63 g protein/kg/day. This value is approximately 25–30% higher than the ones previously computed with indirect methods in women athletes (3). In fact, it is situated within the range recommended for men undertaking a comparable average weekly training volume. The other noteworthy outcome of this study was the variability of individual nitrogen balance that, according to the authors, could be due to the variation in dietary macronutrient composition and meal timing, energy intake, sex-hormone levels, and menstrual cycle phase. All these factors are possible modifying covariates and should be further investigated (21).

## Iron

The critical importance of iron for female athletes is established through its biological role in supporting the function of proteins and enzymes essential for maintaining physical and cognitive performance (22). The best characterized biological role for iron occurs through its incorporation into hemoglobin and myoglobin, proteins responsible for the transport and storage of oxygen. In fact, approximately 65% of body iron is incorporated into hemoglobin, and classical human studies have demonstrated that diminished maximal oxygen consumption occurs as hemoglobin levels decline, affecting endurance performance (23–26). The recommended dietary allowance (RDA) for iron depends on the population and country and is significantly higher for pre-menopausal women (18 mg/day) than it is for men (8 mg/day), mainly due to the regular losses of iron that occur through menstrual bleeding. Female athletes may experience difficulties in consuming the recommended amount of iron per day for a number of reasons, including energy restriction and vegetarianism (27). The bioavailability of iron in vegetarian diets (plant-based foods) is estimated to be about 10%, rather than about 18% from a mixed omnivorous diet. Thus, iron requirements for vegetarians are about 1.8 times higher than for omnivores (28). In addition to the quantity of iron ingested, inadequate composition of the diet may impair iron absorption (deficiencies in vitamin D, copper, meat products or instead excessive consumption of phosphates, phytates, calcium, and tannins) and erythropoiesis (deficiencies in vitamins B12, B6, folic acid, and copper) (29). But a number of factors other than diet may affect iron status in female athletes (22). First, blood losses from menstruation represent the major route of iron excretion in most pre-menopausal women, and female athletes that experience heavy menstrual volume may be at greater risk for poor iron status (30). Other factors that may affect iron status in athletes include losses due to hemolysis or through gastrointestinal bleeding, which may occur following activities such as distance running. Another recently identified mechanism by which athletes, independently of the gender, may experience decreases in iron status occurs in response to inflammation (31). Acute inflammation may arise following strenuous physical activity, and results in increased circulating levels of interleukin-6 (IL-6). IL-6 stimulates the expression of hepcidin (31), a hormone regulator of iron homeostasis, which affects iron absorption as well as export from both enterocytes and macrophages, resulting in functional deficits in iron status. Acute elevations in hepcidin have been demonstrated in a number of studies with athletes and other populations participating in heavy physical activity. In the initial study describing the hepcidin response to exercise, Roecker et al. observed increased IL-6 and urinary hepcidin levels in women following a marathon (32). More recently, elevated hepcidin has been observed in blood of athletes following various forms of exercise (33, 34).

Countermeasures for preventing and treating iron deficiency and iron deficiency anemia have been studied quite extensively. For female athletes not experiencing iron deficiency or iron deficiency anemia, a conventional diet that includes highly bio-available sources of iron, such as meat, seafood, or legumes, coupled with foods rich in iron absorption enhancers, such as ascorbic acid, may be sufficient for maintaining iron status (22). For individuals that have been diagnosed with iron deficiency or iron deficiency anemia, the use of iron fortified foods, iron supplements, or iron injection may be considered (35). Important to note is that in athletes not experiencing iron deficiency, iron supplementation does not increase athletic performance, contrary to the popular idea. Only athletes suffering from iron deficiency may benefit from iron supplementation, preferentially under the most bio-available forms iron sulfate, iron gluconate, or iron fumarate (36). In summary, iron is a key mineral for performance in women athletes. Iron status should therefore be regularly tested and diet should be adapted accordingly. If needed supplements can be taken as iron content is rather limited in food and bio-availability can be impaired by many variables (28).

## Vitamin D and Calcium

Vitamin D and calcium play an important role in an athlete’s health, training, and performance (37). In addition to the well known effects of calcium and vitamin D on bone health, recent research has highlighted non-skeletal benefits, particularly for vitamin D, which include immune and muscle functions, as well as sport performance (37). Vitamin D can be synthesized endogenously by exposing the skin to UVB radiation of the sun but it can also be obtained from the diet. For example, fatty fish and sun-dried mushrooms are good sources of vitamin D. The fractional absorption of vitamin D is approximately 50% except in individuals with malabsorption syndromes (38). Studies over the last 15 years have found that an average vitamin D intake ranges from 100 IU to close to 250 IU/day in athletes over the world. This falls short of the recommended intake of most counties, which varies from 300–400 IU/day in western countries to 1500–2000 IU/day where the sun is less present (39). Definitive thresholds for vitamin D status have not yet been established scientifically. The current cut off for vitamin D deficiency is a serum 25(OH)D concentration below 50 nmol/l and for vitamin D insufficiency, a concentration below 75–80 nmol/l (40, 41). Available data suggest that the prevalence of vitamin D insufficiency in female athletes may range between 33 and 42%, depending on the type of athlete, season, and latitude (42). The high prevalence of vitamin D deficiency is not surprising as there are very few good dietary sources of vitamin D, and thus mean intake is low (43). Additional factors that contribute to diminished vitamin D status in athletes include reduced sun exposure for indoor sports, or reduced production of vitamin D in response to sun exposure due to the use of sunscreen or season of outdoor activity (22). Because of the limited amount of foods containing vitamin D, most athletes will have to rely on other sources to reach RDA for vitamin D such as regular supplementation, reasonable sun exposure, or a combination of dietary intake, sun exposure, and supplementation. Regular consumption of food enriched in vitamin or a daily multivitamin cocktail alone is likely not sufficient to keep a serum concentration above 75–80 nmol/l in the absence of UVB exposure. Taking supplements of vitamin D containing 5000 IU/day for 8–12 weeks may help to reach these levels (42).

Contrary to vitamin D and iron, clear biochemical indices or serum markers of acute calcium intake or calcium status are lacking. Although easily measured in serum, calcium levels are not correlated with acute calcium intake except if the latter is severely restricted (37). The only way to assess calcium status is by dietary assessment. Unfortunately, the precision of this assessment is poor due to the variability of calcium content in food, the lack of information about calcium content in certain foods, and the difficulty for some athletes to report correctly and accurately what they eat. Recommended dietary calcium intake differs somewhat according to the country and age, ranging between 700 and 1000 mg/day for adults and between 700 and 1300 mg/day for post-menopausal women and men above 65 years (37). Although research in athletes is quite scarce, recent reports show that male athletes on average consume more calcium than female athletes, with large inter-individual variability. Some athletes, principally men, consume well above the recommended amount. On the other hand, athletes looking for a low body weight, amongst which distance runners, may have substandard intakes (37). Calcium requirements do not seem to be dependent on the amount of physical activity. Nevertheless, it has been proposed that exercise may increase calcium loss in sweat and urine. As high concentrations of calcium in sweat (~45 mg/l) have already been observed (44), athletes sweating heavily during prolonged training may have augmented calcium loss and elevated requirements. Amenorrheic athletes as well may require an additional 500 mg/day of calcium to keep calcium balance within recommended limits. Although evidence is quite limited, the higher calcium requirement in amenorrheic athletes could be due to inadequate estrogen levels (45). For eumenorrheic athletes, calcium requirements can be reached by integrating several portions of dairy products or five to eight portions of vegetal sources in the daily nutrition. Calcium bioavailability of cow milk is very high with a fractional absorption of 32% (46) but most plant foods often score as well or even better than cow milk (37). Well absorbable plant-based calcium can be found in low-oxalate green leafy vegetables (e.g., lettuce, celery), fortified juice, soy milk or meat, rice milk, and certain legumes (e.g., soybeans, peanuts) (47). Sardines are also an excellent non-dairy source of absorbable calcium. Athletes not meeting their calcium intake may consider calcium supplementation combined with vitamin D, if needed, as a short-term option. However, they should try to increase the daily consumption of calcium-rich foods as part of a well-balanced diet that is adequate in phosphorus, protein, magnesium, and vitamins A and K (37). In case of amenorrhea, calcium requirements are elevated and often not satisfied by daily nutrition alone. Calcium supplementation of 1000 mg/day, preferentially taken in two doses of 500 mg, may in that case, be particularly helpful (37). Calcium carbonate and calcium citrate are well-absorbed sources used in supplements (48). The duration of the supplementation depends on the severity of the deficiency but extends over one or several years. Of note, the effects of calcium supplementation on bone mineral density are short-lived after cessation (49), underlying the importance of long-term strategies such as privileging calcium-rich food in the diet.

## Hydration

The recommended daily amount of water differs somewhat from one country to the other. In the United States, the adequate intake for total water is 3.7 l/day for men and 2.0 l/day for women and in Europe 2.5 l/day for men and 2.0 l/day for women (50). Being particularly active, being exposed to stressful environmental conditions such as heat or altitude, or losing liquid through vomiting or diarrhea may increase daily fluid requirements. For general recommendations for fluid replacement during exercise and specific environmental conditions, the reader is referred to previously published reviews (50–52). However, some specific considerations should be taken into account when adapting those general rules to women. They usually have lower sweating rates than men, essentially due to smaller body mass and lower metabolic rate during physical activity (51). Nevertheless, this difference disappears when sweating rates are reported to body surface area, at least in temperate and hot-dry conditions (53). In hot-wet conditions, women sweat less than men (53), thereby losing less fluid and reducing the risk for hypohydration (50).

Renal handling of water and electrolytes is different between men and depends on the phase of the menstrual cycle. For example, in response to a water load, women have a higher rate of water turnover, particularly during the luteal phase of the menstrual cycle (54). Also, estrogens and progesterone increase renal water and electrolyte retention in resting conditions (55, 56). Finally, body core temperature is increased by up to 0.6°C during the luteal phase. Importantly, despite these hormonal regulations, no indication exists to adapt hydration strategies after exercise according to the phase of the menstrual cycle as the latter does not seem to significantly impact renal water and electrolyte retention during fluid replacement after exercise (57).

In addition, women are more susceptible to hyponatremia compared with men (58). After the Boston Marathon in 2002, 13% of the tested participants had hyponatremia defined as a serum sodium concentration below 135 mmol/l and 0.6% had critical hyponatremia with a serum sodium concentration below 120 mmol/l (59). Among all women tested, 22% were hyponatremic at the end of the marathon, which was approximately three times more than the prevalence in men (8%). Similar proportions were found after the 1997 New Zealand Ironman triathlon where hyponatremia was three times more frequent in women than in men, with an overall prevalence of 18% (60). According to the sample or subjects studied and the weather conditions during the race, the prevalence of hyponatremia can reach up to 29% (58). Interestingly, this prevalence is similar to or even a bit lower than the prevalence of hypohydration and hypernatremia after long-lasting running events (61). To the best of our knowledge, the prevalence of hypohydration and hypernatremia is not different between male and female runners. The higher prevalence of hyponatremia in women is mainly due to the fact that they have a smaller total body water content and extracellular fluid volume, which consequently require less overdrinking than men to dilute serum sodium concentration (50, 62). A variety of psychosocial and/or biological factors may be involved. It has been shown that women overdrink relative to body weight compared to men (63). Other factors that may increase a woman’s risk for overdrinking include longer race times (59) and elevated estradiol and progesterone levels as observed during the luteal phase (62). Women should therefore pay particular attention to the adequate amount of water (0.4–0.8 l/h) they drink during long-lasting events and favor sport drinks containing 4–8% carbohydrates and an appropriate cocktail of electrolytes (1, 50–52). This recommendation is quite broad as sweating rates and sweat electrolytes content vary largely amongst individuals. Athletes should try not to lose more than 2% of their body weight during a race (51).

## Contraceptives

Oral contraceptives typically reduce menstrual blood losses by about 60%, meaning that the iron RDA for women who use oral contraceptives would be 11 mg/day rather than 18 mg/day (28). Although less straightforward than for iron metabolism, it seems that the chronic use of oral contraceptives may also alter carbohydrate and lipid metabolism (64). The use of contraceptives has been shown to increase the availability of free fatty acids at rest and during mild exercise, suggesting a preference for lipid metabolism by skeletal muscle (65). Yet, during heavy exercise, similar decrements in free fatty acids along with similar increments in lactate in oral contraceptives users and normally menstruating women probably reflect an unimpaired carbohydrate metabolism during heavy exercise (65). Those results suggested that oral contraceptives users could benefit from spared glycogen and increase performance when glycogen is a limiting factor (64). Indeed, lower blood glucose levels and a lower total amount of carbohydrate use during prolonged submaximal exercise were measured in eight oral contraceptive users compared with eight controls. Those results suggest that contraceptives users preserve their carbohydrate reserves better than non-users during prolonged exercise and could thereby extend time to fatigue (66). However, this glycogen sparing effect in oral contraceptive users was not confirmed by others, nor was any effect found on hemoglobin, growth hormone, ventilation, lactate, maximal heart rate, and maximal RER (67–69). The previous results suggest that an unknown cellular mechanism could be involved in the lower amount of carbohydrates used during exercise by oral contraceptives users (4). Further investigation is needed to better understand the effect of oral contraceptives on carbohydrate and fat metabolism at rest and during exercise and the potential implication this would have on nutritional strategies for the pill users. Finally, the use of oral contraceptives might also influence the amount of liquid ingested daily as estrogens and progesterone increase renal water and electrolyte retention in resting conditions (55, 56). Although acute replacement of exercise-induced fluid losses is not affected by the normal menstrual cycle (57), no study specifically looked at fluid replacement after exercise in long-term oral contraceptive users.

## Racing Dietary Advice

Nutritional recommendations for female distance runners have long been a simple extrapolation from male athletes, somewhat adapted to their smaller size (1), but neglecting the fact that women oxidize more fat and less carbohydrate than men during endurance exercise (70). The efficacy of a carbohydrate loading phase has therefore been tested in women and compared to men. In a first study, increasing dietary carbohydrate intake from 55 to 75% of habitual energy intake for four days increased glycogen storage and enhanced cycling performance in male but not in female athletes (71). The authors attributed this lack of effect to the low total energy intake of the female subjects that restricted carbohydrate amounts to less than 6.5 g/kg/day to comply with the protocol, namely 75% of habitual energy intake. In a follow-up study from the same research team, an additional group was added in which 75% of a higher total energy intake achieved carbohydrate amounts above 8 g/kg/day (72). With more carbohydrate and extra energy, women increased muscle glycogen content, although the level achieved was somewhat lower than in men (72). It appears therefore that female athletes can benefit from carbohydrate loading only if they are ready to consume adequate energy and carbohydrate. During the race, the dietary recommendations for men and women regarding the use of sport drink (73, 74) (see also previous paragraph) and for post-exercise glycogen re-synthesis (75) seem to be similar. Importantly, in a study looking at the effect of menstrual phase on carbohydrate loading and utilization, no effect of phase on substrate utilization during exercise was observed (76). However, confirming previous findings (77), lower resting glycogen levels were measured in the mid-follicular than in the mid-luteal phase of the menstrual cycle in endurance athletes, which could fortunately be overcome by carbohydrate loading (76). All together, carbohydrate loading seems a useful strategy in women even though the response might be a little bit less advantageous for performance in women than in men.

## Conclusion

Female endurance runners usually care about their nutrition but the low energy intake generally encountered in this population may lead to nutritional imbalances and deficiencies. Reaching the minimal value of energy intake of 45 kcal/kg of fat free mass/day plus the amount of energy needed for physical activity and this together with a well-balanced diet should improve the nutritional status of many female runners. Consequently, a positive impact on their general health status can be expected.

Practical recommendations

- Energy intake should be above 45 kcal/kg fat free mass and additional energy intake should cover energy expenses during physical activity.
- Daily protein intake should be between 1.2 and 1.6 g/kg/day.
- Although less responsive to carbohydrate loading than their male counterpart, female runners can retrieve some benefits from ingesting >8 g carbohydrates/kg/day before a competition.
- Female runners should be particularly aware of the high risk of being deficient for iron, calcium, and/or vitamin D. Recommended daily allowances are 18 mg for iron, 1000 mg for calcium, and between 300 and 2000 IU for vitamin D, the latter being inversely related to sun exposure.

## Conflict of Interest Statement

The authors declare that this work was conducted in the absence of any commercial or financial relationships that could be construed as a potential conflict of interest.
